# Seasonal and Longitudinal Changes in Body Composition by Sport-Position in NCAA Division I Basketball Athletes

**DOI:** 10.3390/sports6030085

**Published:** 2018-08-22

**Authors:** Jennifer B. Fields, Justin J. Merrigan, Jason B. White, Margaret T. Jones

**Affiliations:** 1 Frank Pettrone Center for Sports Performance, George Mason University, Fairfax, VA 22030, USA; jmerrig2@gmu.edu (J.J.M.); jwhite35@gmu.edu (J.B.W.); mjones15@gmu.edu (M.T.J.); 2 Division of Health and Human Performance, George Mason University, Manassas, VA 20110, USA

**Keywords:** body fat, collegiate athletes, fat free mass, women athletes

## Abstract

The purpose of this study was to assess the body composition of male and female basketball athletes (*n* = 323) across season, year, and sport-position using air displacement plethysmography. An independent sample *t*-test assessed sport-position differences. An analysis of variance was used to assess within-subjects across season (pre-season, in-season, and off-season), and academic year (freshman, sophomore, and junior). For both men and women basketball (MBB, WBB) athletes, guards had the lowest body fat, fat mass, fat free mass, and body mass. No seasonal differences were observed in MBB, but following in-season play for WBB, a reduction of (*p* = 0.03) in fat free mass (FFM) was observed. Across years, MBB showed an increase in FFM from freshman to sophomore year, yet remained unchanged through junior year. For WBB across years, no differences occurred for body mass (BM), body fat (BF%), and fat mass (FM), yet FFM increased from sophomore to junior year (*p* = 0.009). Sport-position differences exist in MBB and WBB: Guards were found to be smaller and leaner than forwards. Due to the importance of body composition (BC) on athletic performance, along with seasonal and longitudinal shifts in BC, strength and conditioning practitioners should periodically assess athletes BC to ensure preservation of FFM. Training and nutrition programming can then be adjusted in response to changes in BC.

## 1. Introduction

Body composition (BC) plays a critical role in athlete health and sport performance. Extreme levels of body fat (BF%) may bring about severe health consequences. Low BF% has been related to decreased bone density, menstrual dysfunction, and disordered eating habits; high BF% has been related to the onset of cardiovascular disease risk factors. Generally, lesser amounts of fat mass (FM) coupled with greater amounts of fat free mass (FFM), particularly muscle mass, are favorable for athletes [1] and provide the basic foundation for sport-specific technical skills and locomotor activities [2]. The specific balance of FM and FFM, or overall BF%, may be dependent upon sport-position. For example, when evaluating BC across positions in collegiate and elite level basketball players [3,4,5], guards were reported to be smaller-bodied with lower BF% and FM when compared to centers and forwards. Yet, prior studies have lacked sufficient sample sizes, which allowed for enhanced generalizability and a better understanding of sport-position BC measures, as well as possible performance evaluations. 

In addition, routine monitoring of BC in athletes is important to evaluate health assessments, track changes, and make necessary adjustments to a diet or training program [6]. Despite the health and performance implications of BC, few studies have examined both seasonal and longitudinal BC changes in National Collegiate Athletic Association Division I (NCAA DI) men and women basketball (MBB, WBB) athletes. 

Consistent and effective monitoring of BC across basketball seasons and years may provide coaches with beneficial feedback in regard to evaluation of strength and conditioning programs and athlete diets. Ultimately, this will enable coaches to make specific adjustments to achieve and maintain optimal BC measures. Prior investigations examining seasonal BC changes in MBB athletes are minimal [7,8], and the few published studies provide contrasting results. Groves et al. (1993) reported a reduction in BF% and BM from pre-season to off-season for NCAA MBB athletes (*n* = 8), while Hoffman et al. (1991) found no change in BF% or BM from pre-season to off-season in MBB athletes (*n* = 9). Both studies had limited sample sizes and used skinfolds to predict BF% [7,8]. While also limited, seasonal BC reports in WBB have shown a reduction in BF% from pre- to post-season [1,9,10]. These limited results warrant the need for further research relative to basketball athletes regarding seasonal BC change.

Furthermore, a unique component of our study was the longitudinal assessment of BC. Few studies have examined BC changes across years in collegiate MBB and WBB athletes [1,11], highlighting the need for additional research that investigates these BC changes. Therefore, the primary purpose of the current study was (1) to provide descriptive data across sport and sport-position in NCAA-DI MBB and WBB athletes; (2) to examine seasonal changes in BC measures; and (3) to document yearly changes in BC measures. We hypothesized that guards would be smaller and leaner compared to forwards, and that MBB and WBB would show reductions in BF% from pre- to post-season, while gaining FFM across years.

## 2. Materials and Methods

### 2.1. Subjects

NCAA DI men and women basketball players, aged 18–24, participated in the study. All athletes were under the direction of a strength and conditioning coach and were following sport-specific training regimens with neuromuscular demands particular to their respective sport and training program. Furthermore, nutritional programming was provided by George Mason University’s basketball sports dietitian. All participants completed a medical history form and had been cleared previously for intercollegiate athletic participation. Risks and benefits were explained to athletes and an institutionally approved consent form was signed prior to participation. The Institutional Review Board for Human Subjects approved all procedures.

### 2.2. Procedures

In order to obtain sport-position specific BC data, body composition was assessed over an eight-year period for MBB and a nine-year period for WBB (MBB, *n* = 127; WBB, *n* = 196) using air displacement plethysmography at one-time point per athlete. The MBB and WBB athletes (MBB, *n* = 16; WBB, *n* = 29) that completed three BC assessments within the same year (pre-season: November; in-season: March; off-season: July) were included in a secondary analysis to assess seasonal changes. Furthermore, those who completed BC assessments (MBB, *n* = 14; WBB, *n* = 8) across three consecutive years (freshman, sophomore, and junior) were included in an analysis of longitudinal BC changes. Longitudinal measurements were separated by a 12-month period. Differences in BF%, FM, FFM, and BM were evaluated. Findings were compared across sport-position, seasons, and years.

Athletes were instructed to refrain from exercise, eating, and drinking for ≥2 h prior to testing. The majority of testing was conducted early in the morning following an overnight fast. Upon arrival to the laboratory, subjects’ body mass was recorded to the nearest 0.01 cm and 0.02 kg, respectively, using a stadiometer (Detecto, Webb City, MO, USA) and electronic scale (BOD POD; COSMED USA Inc., Concord, CA, USA) calibrated according to manufacturer guidelines. Body composition was assessed using air displacement plethysmography (BOD POD, model 2000A; COSMED USA Inc., Concord, CA, USA), which has been shown to be a reliable and valid method for measuring BC [12]. Prior to each testing session, calibration procedures were completed using an empty chamber and a calibrating cylinder of a standard volume (49.55 L), according to the manufacturer guidelines. Participants were instructed to wear a formfitting sports bra (women), spandex shorts, and swim cap, and to remove all jewelry, in accordance with standard operating procedures that reduced excess air displacement. A trained technician performed all testing. Two tests were performed to ensure reliability of the assessment. If the tests results were not within 150 mL of each other, two additional tests were administered. Our lab’s test to test reliability of this body composition assessment yielded high reliability for body mass (*r* = 1.0), body fat percent (*r* = 0.997), and fat-free mass (*r* = 1.0) [13]. 

### 2.3. Statistical Analysis

SPSS version 25.0 (IBM, Armonk, NY, USA) was used for data analysis. To test for significant differences across sport-position, we used an independent samples *t*-test. To assess seasonal (pre-season, in-season, and off-season) and longitudinal (freshman, sophomore, and junior) changes, we used a repeated measures ANOVA. 

## 3. Results

Body compositions between MBB and WBB sport-positions are included in Table 1 (means ± SD; BF%, FM, FFM, and BM). For both MBB and WBB, guards, when compared to forwards, had significantly (*p* < 0.001) less BF% (MBB: 8.6 ± 3.3% vs. 14.9 ± 4.8%; WBB: 19.2 ± 6.3% vs. 24.2 ± 5.7%), FM (MBB: 7.4 ± 3.1 kg vs. 15.9 ± 5.7 kg; WBB: 13.4 ± 5.4 kg vs. 20.5 ± 7.4 kg), FFM (MBB: 77.7 ± 6.4 kg vs. 89.4 ± 7.5 kg; WBB: 54.63 ± 64.4 kg vs. 61.8 ± 6.0 kg), and BM (MBB: 85.2 ± 7.5 kg vs. 105.3 ± 8.1 kg; WBB: 68.0 ± 7.4 kg vs. 82.2 ± 12.5 kg). After adjusting for height, however, FFM differences were no longer apparent (MBB: 22.2 ± 1.8 vs. 22.0 ± 1.4; WBB: 18.6 ± 1.7 vs. 18.6 ± 1.9).

For season phases, there were no significant differences in BF%, FM, FFM, or BM observed in MBB (Table 2). Following in-season play, a significant reduction in FFM (*p* = 0.01) was observed for WBB (March: 58.8 ± 6.5 kg vs. July: 56.9 ± 6.7 kg) (mean difference; 95% CI: −0.814; −1.601, −0.027) (Table 2). 

When analyzing MBB body composition changes across years, FFM increased significantly (*p* < 0.05) from freshman (82.0 ± 8.4 kg) to sophomore year (83.5 ± 8.4 kg) (mean difference; 95% CI: 1.534; 0.008, 3.059) and remained unchanged through junior year (84.1 ± 9.1 kg) (mean difference; 95% CI: 0.618; −0.352, 1.587) (Table 3). For WBB, no differences were observed across years in BM, BF%, and FM. FFM did not increase from freshman to sophomore years (mean difference; 95% CI: 0.918; −0.891, 2.728), but significant (*p* = 0.02) increases occurred from sophomore (56.2 ± 3.9 kg) to junior year (57.7 ± 4.0 kg) (mean difference; 95% CI: 1.448; 0.259, 2.638) (Table 3). 

## 4. Discussion

Previously published body composition data pertaining to sport-position, sport season, and time are limited for NCAA DI men and women basketball athletes. Therefore, our study’s purpose was to contribute descriptive BC data for men and women collegiate basketball athletes, and to evaluate seasonal and longitudinal changes in BC metrics. Researchers hypothesized that (1) guards would be smaller and leaner compared to forwards; and (2) MBB and WBB would show reductions in BF% from pre- to post-season, and gain FFM across years. 

In the current study, MBB and WBB guards were significantly smaller and leaner than forwards, which is in support of previously published research [3,4,5]. In basketball, a player’s size largely determines the position played on the team [5]. For example, it is advantageous to assign the largest players positions closer to the basket, which enhances shot blocking and rebounding performances. Smaller players, however, are assigned perimeter positions that facilitate moving the ball quickly down the court [14]. The smaller physique is suitable for guards, as the position requires speed and agility skills, an ability to rapidly transfer the ball from defense to offense, and an ability to defend against the quickest players on the opposing team [5,14].

The investigation of BC changes across sport seasons in collegiate basketball athletes is not extensive. In the current study, MBB demonstrated no significant changes in BF%, FM, FFM, or BM from the initiation of pre-season to completion of the in-season. These findings are similar to previous findings in a smaller sample of NCAA D1 MBB athletes (*n* = 9) [8]. However, results appear inconsistent, as Groves and Gayle observed a significant reduction in BF% from the start of pre-season (October) to conclusion of the regular season (April) [7]. Similarly, elite junior MBB athletes have shown a 3.7% and 2.5% increase in FFM and BM over sport seasons, with no change in absolute FM [15]. However, the small sample sizes (*n* = 5) makes generalizing results difficult [15]. Furthermore, each of these studies utilized different BC measurement assessments (i.e., skinfolds and doubly labeled water). Therefore, we recommend that caution be exercised when comparing results from different measurement techniques [16]. 

In the current study, WBB athletes showed a decrease in FFM following the in-season period, while no changes were observed in BF%, FM, or BM. These results are in contrast with previously reported findings, where WBB athletes (*n* = 38) displayed decreases in BF% from the pre- to post-season, ranging from −0.8% to −1.4% [1,9,10], and increases in FFM and BM in elite junior WBB players (*n* = 9) [15]. The reduction in FFM observed in the current study is of concern, as previous literature has shown a correlation between FFM and bone mineral density [17], strength [18,19], speed [20], and power [18,21]. Reductions in FFM across seasons were also observed in NCAA Division I collegiate softball athletes [17], thus signifying an ongoing concern for women athletes in regard to maintaining FFM.

When analyzing longitudinal data, MBB athletes displayed an increase in FFM from years one to two with no change from years two to three. No other changes in BC were observed. Although a lack of published findings makes comparisons challenging, prior research in collegiate MBB players (*n* = 16) showed an increase in FFM and BM from years one to two (+2.5 ± 3.8 kg, *p* < 0.05) (*n* = 19) [22], and years three to four (+2.8 ± 3.4 kg, *p* < 0.01) [21]. BM also increased in a similar pattern, from years one to two (+2.4 ± 3.0 kg, *p* < 0.01), and years three to four (+3.7 ± 2.9 kg, *p* < 0.01) [22]. The largest change observed between years one and two is likely due to athletes following a structured strength training regimen that they later became adapted to [22,23]. 

Although data on basketball is limited, longitudinal BC responses have been widely examined in NCAA football athletes [24,25,26]. Division I wide receivers and defensive backs have shown steady increases in BM, with the largest increases observed from years one to two (82.65 ± 3.42 kg to 87.42 ± 2.75 kg; 7.7% increase) [25,26], and no reported changes in BF%. Linemen have also shown strong increases in BM from years one to two (1.9% gain) [24,25,26], as well as increases in lean body mass (LBM), growing from 97.89 kg to 101.09 kg in years one to two, and 101.09 kg to 104.55 kg in years two to three [26]. The different BC responses found between skill positions (receivers and defensive backs) and linemen may be due to the athletic nature of each position. Skilled players have been shown to have the lowest BF%, as these positions require strong speed and agility skills. On the other hand, linemen require greater amounts of strength and rely on higher levels of FFM, thus explaining why increases in such areas were noted over the years. The same conclusions can be speculated about in basketball, where the type of playing strategies made by the team may dictate the likelihood of BC changes across seasons. Teams that play a faster style of game without a true center position may not experience significant changes in body composition, a finding similar to what was reported in football skill positions. 

In the current study, WBB athletes showed no differences across years for BM, BF%, and FM. FFM did not increase from freshman to sophomore year, but a significant increase occurred from sophomore (56.2 ± 3.9 kg) to junior year (57.7 ± 4.0 kg). Few studies have measured longitudinal BC changes; therefore, more research is needed to understand these growth trends over time. Stanforth et al. (2014) observed an increase in BF% (year 1: 25.8 ± 0.8%; year 2: 25.9 ± 0.8%; year 3: 27.5 ± 0.9%) and FM (year 1: 20.0 ± 0.7 kg; year 2: 20.1 ± 0.8 kg; year 3: 21.8 ± 0.8 kg) across three consecutive years in 38 NCAA Division I WBB athletes [1]. Petko and Hunter, however, saw no changes in BF% from freshman to senior year (*n* = 11) (% change: 0.3 ± 0.8%) [11]. Again, this may be due to the various methods used to evaluate BC, training programs, or the style of game play. In the current study, the increases in FFM observed from sophomore to junior year indicate a beneficial adaptation to training, yet it is surprising no changes were observed from freshmen to sophomore year, a period in which new players are commonly exposed to a structured resistance training program. Future research should consider evaluating BC changes across seasons and years with multiple teams utilizing the same measurement tools for analyses. 

In WBB athletes, we found the reduction in FFM across seasons and lack of muscle gain across years a cause for concern. This may, in part, be due to a fear of women appearing overly muscular [27], as well as potential training program differences between MBB and WBB teams. These speculations are based upon the self-reported beliefs of MBB coaches that women are physically inferior to men, lack commitment in the sporting sphere, and are not capable of training with male athletes [28]. Further, high school strength coaches have expressed concern that women are often not challenged to work as hard athletically as men, perhaps because of the perception that women are “dainty” and “ought not to sweat too hard” [29]. If said beliefs are present, regardless of sport level, it is likely that male strength coaches will treat women athletes differently than their male counterparts. These unfounded perceptions are increasingly apparent in high school athletics, where 50% of coaches for men’s sports required their athletes to strength train, and only 9% of coaches for women’s sports did the same [30]. In fact, researchers have suggested that an even greater focus on force production should be utilized when training women for power [30]. To combat losses in FFM, it is recommended that strength training be made a priority in resistance exercise programming for female athletes [30,31]. 

In conclusion, strength and conditioning practitioners must ensure training and nutrition programs are aimed at optimizing muscle development to support performance in basketball athletes, particularly in WBB. Routine monitoring of BC measures may assist in the evaluation of such programs and will allow coaches to make adjustments, as needed.

## Figures and Tables

**Table 1:** Body composition between basketball sport-positions.

Sex	Position	BF (%)	FM (kg)	FFM (kg)	BM (kg)	Height (cm)	FMI (kg/m^2^)	FFMI (kg/m^2^)
Men	Guard (*n* = 68)	8.6 ± 3.3 ^1^	7.4 ± 3.1 ^1^	77.7 ± 6.4 ^1^	85.2 ± 7.4 ^1^	187.4 ± 7.0	2.1 ± 0.9 ^1^	22.2 ± 1.8
	Forwards (*n* = 59)	14.9 ± 4.8 ^2^	15.9 ± 5.6 ^2^	89.5 ± 5.9 ^2^	105.3 ± 8.0 ^2^	201.7 ± 4.0	3.9 ± 1.4 ^2^	22.0 ± 1.4
Women	Guard (*n* = 105)	19.2 ± 6.3 ^1^	13.4 ± 5.4 ^1^	54.6 ± 4.4 ^1^	68.0 ± 7.4 ^1^	171.6 ± 5.0 ^1^	4.52 ± 1.8 ^1^	18.6 ± 1.7
	Forwards (*n* = 91)	24.2 ± 5.7 ^2^	20.5 ± 7.7 ^2^	61.8 ± 5.9 ^2^	82.2 ± 12.5 ^2^	183.5 ± 4.4 ^2^	6.07 ± 2.3 ^2^	18.6 ± 1.9

Values are mean ± SD; BF%: Body fat percent; FM: Fat mass; FFM: Fat free mass; BM: Body mass; FMI: Fat mass index; FFMI: Fat free mass index; Order of significance presented: ^1^ < ^2^; Level of significance set at *p* < 0.0125.

**Table 2:** Body composition measures across basketball season phases.

Sex	Measure	Pre-Season	In-Season	Off-Season
Men (*n* = 16)	BF (%)	10.4 ± 5.2	10.0 ± 4.5	10.7 ± 6.2
	FM (kg)	10.3 ± 6.3	9.7 ± 5.5	10.7 ± 7.5
	FFM (kg)	83.4 ± 9.2	83.6 ± 8.8	83.1 ± 9.2
	BM (kg)	93.7 ± 14.1	93.3 ± 13.0	93.3 ± 14.5
Women (*n* = 29)	BF (%)	16.8 ± 7.4	17.0 ± 7.7	17.7 ± 8.5
	FM (kg)	21.7 ± 6.4	21.6 ± 6.7	22.2 ± 6.7
	FFM (kg)	57.9 ± 7.7 ^1^	58.8 ± 6.5 ^2^	56.9 ± 6.6 ^1^
	BM (kg)	75.4 ± 13.1	75.9 ± 12.8	75.3 ± 13.5

Values are mean ± SD; BF%: Body fat percent; FM: Fat mass; FFM: Fat free mass; BM: Body mass; Order of significance presented: ^1^ < ^2^; Level of significance set at *p* < 0.05.

**Table 3:** Body composition measures across years in basketball athletes.

Sex	Measure	Year 1	Year 2	Year 3
Men (*n* = 14)	BF (%)	14.1 ± 4.2	12.6 ± 4.7	13.4 ± 4.5
	FM (kg)	14.0 ± 5.6	12.5 ± 6.1	13.3 ± 5.8
	FFM (kg)	82.0 ± 8.4 ^1^	83.5 ± 8.4 ^2^	84.1 ± 9.1 ^2^
	BM (kg)	95.9 ± 12.8	96.0 ± 12.5	97.4 ± 12.9
Women (*n* = 8)	BF (%)	21.0 ± 5.5	21.1 ± 5.1	20.5 ± 5.3
	FM (kg)	14.8 ± 4.5	15.3 ± 4.7	15.1 ± 4.9
	FFM (kg)	55.2 ± 3.4 ^1^	56.1 ± 3.9 ^1^	57.7 ± 4.0 ^2^
	BM (kg)	70.1 ± 4.6	71.4 ± 6.5	72.8 ± 6.5

Values are mean ± SD; BF%: Body fat percent; FM: Fat mass; FFM: Fat free mass; BM: Body mass; Order of significance presented: ^1^ < ^2^; Level of significance set at *p* < 0.05.
